# Immunohistochemical Analysis of CD99 and PAX8 in a Series of 15 Molecularly Confirmed Cases of Ewing Sarcoma

**DOI:** 10.1155/2020/3180798

**Published:** 2020-06-17

**Authors:** LM. Chinchilla-Tábora, J. Ortiz Rodríguez-Parets, I. González Morais, J. M. Sayagués, M. D. Ludeña de la Cruz

**Affiliations:** ^1^Department of Pathology and IBSAL, University Hospital of Salamanca, Salamanca, Spain; ^2^Department of Pathology, University Hospital of Salamanca, Salamanca, Spain

## Abstract

Ewing sarcomas are an uncommon group of malignant neoplasms. A multidisciplinary approach is highly recommended to reach a correct diagnosis, considering the clinical, radiological, and histopathological aspects. Since in up to 90% of cases, the translocation t (11; 22) (q24; q12) occurs resulting in a chimeric fusion transcript EWSR1-FLI-1. The pathologist has several tools in addition to conventional techniques (hematoxylin and eosin), such as immunohistochemistry, which plays a very important role in the differential diagnosis. We present a series of 15 cases of molecularly confirmed ES, in which we found a sensitivity of 100% for CD99 and 80% for PAX8 by immunohistochemistry. This indicates a high sensitivity; however, it is known that both CD99 and PAX8 are also expressed in other tumours. Therefore, molecular confirmation should be performed in all cases.

## 1. Introduction

Ewing sarcomas (ESs) are relatively uncommon and represent at least 6% of primary malignant bone tumours. It is the second most common sarcoma of the bone in children and young adults after osteosarcoma [1, 2].

There are currently several tools to approach the diagnosis of ES. A correct anamnesis and physical examination of the patient looking for signs or symptoms such as a rapidly growing mass mainly in the extremities, pain, and intermittent fever should be performed. The imaging techniques such as computed tomography (CT) and magnetic resonance imaging (MRI) are essential to determine the size, the osteolytic or sclerotic signs, the extension, and ill-defined limits of these tumours. MRI is also vital to evaluate response to neoadjuvant therapy, direct surgical resection, and detect local recurrence or metastatic disease [3]. The histological and immunohistochemical characterization of the tumour are essential for a correct histopathological diagnosis. The genetic study by the reverse transcriptase-polymerase chain reaction (RT-PCR) or fluorescent in situ hybridization (FISH) shows that, in approximately 90% of cases, a highly sensitive chromosomal translocation exists: t (11; 22) (q24; q12). The fusion of the EWSR1 gene on 22q12 with the *FLI1* gene on 11q24 results in a chimeric fusion transcript EWSR1-FLI-1 [4]. In the current era, with the advent of next-generation sequencing (NGS) technology, the electron microscopy studies for the ES diagnosis become practically obsolete; however, they can be useful to identify varying degrees of neuroectodermal differentiation.

Immunohistochemistry (IHC) is a well-established tool, which is widely used to help identify a wide spectrum of specific pathological processes. It is also used in experimental research involving the bone and soft tissue. Besides descriptive analyses, multiparametric, semiquantitative scoring systems for evaluating different bone parameters represent a universal approach to include histopathologic information in biomedical research [5, 6].

CD99 represents a surface glycoprotein encoded by the pseudo-autosomal MIC2 gene located in the short arm of the X and Y sex chromosomes [7]. CD99 (MIC2) has been widely analysed in the ES by different authors [8, 9]. Strong and diffuse membranous expression is seen in approximately 95% of ES [10]. CD99 is a nonspecific marker for ES, but it is a very sensitive marker [11]. It is known that many other small round cell tumours can show a mild, focal, and irregular CD99 immunoreactivity as anaplastic large-cell lymphoma, lymphoblastic lymphoma/leukaemia [12, 13], poorly differentiated synovial sarcoma (round cell variant) [14, 15], between 10% and 25% of rhabdomyosarcomas [16], and approximately 20% of desmoplastic small round cell tumour [17]. Rhabdomyosarcomas and desmoplastic small round cell tumour show a cytoplasmic CD99 immunoreactivity while in ES is typic the membranous pattern of immunoreactivity.

Paired box gene-8 protein (PAX8) is a nephric-lineage transcription factor and is a crucial transcription factor for organogenesis of the thyroid gland, kidney, and müllerian system [18]. Its expression has also been described commonly in epithelial tumours of the thyroid gland, parathyroid glands, kidney, thymus, and female genital tract, most commonly in serous ovary carcinoma and endometrioid tumours [19].

Several studies have been published over the PAX8 expression in sarcomas such as rhabdomyosarcoma, malignant rhabdoid tumour, and clear cell sarcoma of the kidney [20]. Expression of PAX8 has been reported by Chang et al. in 1 of 27 cases of ES/primitive neuroectodermal tumour (PNET) [21].

We present a series of 15 cases of molecularly confirmed ES, trying to find the meaning of its immunoreactivity for CD99 and PAX8, to determine whether there is enough sensitivity to be useful in the diagnosis of these tumours.

## 2. Materials and Methods

We have done a retrospective study of 15 cases diagnosed as ES in our department in the last six years (2014–2019).

The inclusion criteria applied were [1] previous radiological characterization of the tumour and [2] availability of adequate 10% neutral buffered formalin-fixed paraffin-embedded (FFPE) blocks of tumoral tissue for IHC studies. Clinical and radiological data analysed for this review are compiled in Table 1. Clinical and radiological data were obtained from the reports collected in the database used in our institution (electronic medical record).

Once obtained the tumoral tissue as trephine biopsy, core needle biopsy, or resection and after being processed in our laboratory of pathology (10% neutral buffered formalin-fixed during at least 12 hours and then paraffin-embedded), a battery of hematoxylin and eosin (H&E) stained slides was prepared in all cases (Dako-Agilent, CoverStainer).

We designed an immunohistochemical panel that includes CD99 and PAX8 (Table 2). Both histological and immunohistochemical analyses were evaluated using a Leica DM2000 LED light microscope.

At the postanalytical stage, we applied a combined multiparametric, semiquantitative IHC scoring system, the immunoreactive score (IRS), which consists in a range of 0–12 as a product of multiplication of the positive cell proportion score (0–4) and the staining intensity score (0–3), resulting in one of the next: 0-1(negative), 2-3 (mild), 4–8 (moderate), and 9–12 (strongly positive) [22, 23], as shown in Table 3.

Fluorescence in situ hybridization (FISH) using a Dual Color Break Apart Probe (Vysis EWSR1 Break Apart FISH Probe Kit), to detect the rearrangement of the EWSR1 gene, was performed in all cases. 3 *μ*m sections of FFPE tumoral tissue were obtained from all the cases. A battery of H&E stained slide interlayer with unstained slides was prepared to identify the tumoral areas. Slide pretreatment for FISH was performed according to the standard recommendations by the manufacturer. The images were captured with a Leica DM 5500 B fluorescent microscope with a charge-coupled device camera (Leica Biosystems). The images were analysed by both a pathologist and a biologist using CytoVision software. At least 100 cells were scored for each slide. The presence of EWSR1 was reported only when >20% of the tumour cells showed split signals (separation of signals by more than 2 signal diameters).

## 3. Results

### 3.1. Clinical Results

Table 1 contains the clinical details of the 15 cases described herein together. The age range was between 14 and 73 years with a mean of 31 years, a median of 24 years, and a mode of 14 years. 60% of patients were younger than 30 years. We found male predominance (66.66% males and 33.33% females), with a sex ratio of 2 : 1. The most prevalent site of involvement with 46.66% (7 cases) was the metaphyseal-diaphyseal portion of long bones. The other 53.33% (8 cases) involved the soft tissue from the perineal, gluteus, thigh, and retroperitoneum. One case involved the chest wall and lung (Askin tumour), and two cases were pulmonary metastasis.

### 3.2. Radiological Results

In the 15 cases (100%), a radiological study with CT and MRI was performed before the biopsy procedure. After that, a complete extension study with positron emission tomography-computed tomography (PET-CT) was performed. The radiological size of the tumours ranges from 4 cm to 18 cm in the greatest diameter, with a mean of 10 cm. 8 cases (53.33%) presented bone tissue involvement by tumour at the time of diagnosis, and 100% of them showed osteolytic signs in the radiological study. The other 7 cases showed ill-defined limits in the radiological study, as shown in Table 1.

### 3.3. Histological Results

**Figure 1:**
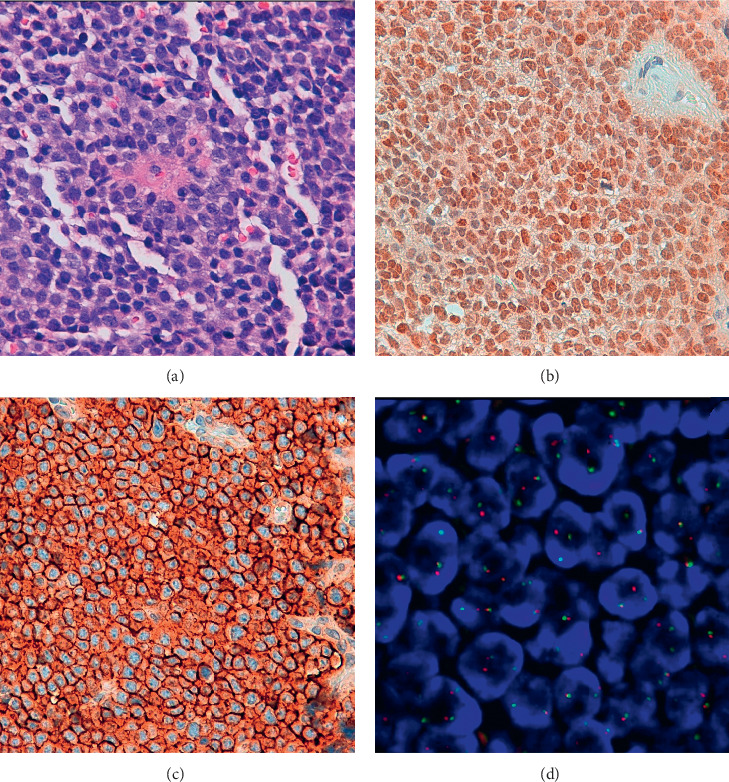
(a) Uniform small round tumour cells with round nuclei and scanty cytoplasm (H&E). (b) Moderate nuclear immunoreactivity for PAX8 (IHQ). (c) Strong and diffuse membranous immunoreactivity for CD99 (IHQ). (d) Split signals due to the rearrangement of EWSR1 (FISH).

A small variability of histological patterns was found among all the cases. The vast majority of cases was composed of a uniform small and round cell with clear or lightly eosinophilic periodic acid-Schiff- (PAS-) positive cytoplasm and indistinct cytoplasmic membranes. The tumoral cells contained rounded nuclei with fine and homogeneous chromatin. Some cases showed areas with neuroectodermal differentiation (arrayed in rosettes as Homer Wright rosettes), as shown in Figure 1(a).

### 3.4. Immunohistochemical Results

In twelve cases (80%), PAX8 showed a nuclear mild to moderate pattern of immunoreactivity (Table 4 and Figure 1(b)). All cases (100%) showed membranous diffuse and strong immunoreactivity for CD99 (Table 3 and Figure 1(c)).

### 3.5. Molecular Results

EWSR1 gene rearrangement was present in all cases (100%) in different percentages of tumoral cells (Table 4 and Figure 1(d)).

## 4. Discussion

ES represent at least 6% of primary malignant bone tumours being more common in children and young adults [1, 2]. We report a serial of 15 molecularly confirmed cases of ES.

In the group of patients analysed for this review, the age range was between 14 and 73 years with a mean of 31 years and a median of 24 years [24]. These results are similar to the age range found by other authors but also can affect older patients as the results published by Marilena Cesari and coworkers. They identified thirty-one patients with ages ranging from 40 to 70 years (median 45 years) [25].

The three bone regions most frequently affected by ES are the pelvic bones, especially the ilium, the diaphysis, or the metaphyseal-diaphyseal portion of long bones (femur, tibia, and humerus), and the chest wall (ribs). Radiologically, the lesions may be lytic, mixed lytic-sclerotic, or, rarely, predominantly sclerotic. CT and particularly MRI imaging are invaluable in further delineating the extent of disease not readily manifested on plain radiographs. Gallium scintigraphy and gadolinium-enhanced MRI images are the best for following the response to therapy [26]. A radiological study with CT and MRI was performed before the biopsy procedure in the 15 cases (100%) analysed in this work. After that, a complete extension study with the PET-CT scan was performed in all of them. Eight of the 15 cases (53.33%) showed osteolytic damage in the radiological study.

Immunohistochemistry is still nowadays a useful tool for pathologists and oncologists because it gives information related to the tumoral oncogenesis. Immunohistochemistry is crucial for the differential diagnosis in metastatic cases of unknown origin. It can also be useful as a biomarker providing information about the susceptibility to specific therapies and helping us to determine the prognostic and therapeutic factors [27].

We obtained similar results with respect to the immunoreactivity of CD99 in ES compared with other authors. Strong, diffuse membranous expression is seen in approximately 95% of ES [10]. CD99 is a nonspecific marker for ES, but it is a very sensitive marker [11]. In this way, Baldauf and coworkers proposed a model of workflow to establish a diagnosis of ES: in the case of clinically and/or radiologically suspected Ewing sarcoma, a biopsy should first be stained for CD99. If CD99 is positive (defined as IRS > 2), confirmatory molecular diagnostic procedures (such as FISH, RT-PCR, and/or NGS techniques), if available, are preferred [28]. This is necessary to rule out other round cell tumours that can show CD99 immunoreactivity in the differential diagnosis. The molecular techniques such as FISH and RT-PCR can detect the rearrangement of the EWSR1 gene, but they are not exclusive for ES. EWSR1 gene has been identified as a partner in a wide variety of clinically and pathologically diverse sarcomas as well as some nonmesenchymal tumours. The former include ES and similar (Ewing-like) small round cell sarcomas, desmoplastic small round cell tumour, myxoid liposarcoma, extraskeletal myxoid chondrosarcoma, angiomatoid fibrous histiocytoma, clear cell sarcoma of soft tissue and clear cell sarcoma-like tumours of the gastrointestinal tract, primary pulmonary myxoid sarcoma, extrasalivary myoepithelial tumours, and sporadic examples of low-grade fibromyxoid sarcoma, sclerosing epithelioid fibrosarcoma, and mesothelioma [29]. To confirm the diagnosis of ES, it is necessary to detect the exact fusion partner of the EWSR1 gene by FISH, RT-PCR, or NGS. Approximately 85% of patients with ES have EWSR1-FLI1 fusions; EWSR1-ERG fusions are present in 10% of cases, whereas in 3% of cases, fusions between EWSR1 and other members of the ETS family of transcription factors are detected [30].

The immunoreactivity of PAX8 in ES has not been widely studied. Bui et al. had investigated the PAX8 staining pattern of primary renal and extrarenal ES/PNET to explore its potential diagnostic and prognostic role. They also have found a moderate (2+) and strong (3+) immunoreactivity for PAX8 in two cases of primary renal ES, respectively, and 14 of 22 (64%) extrarenal ES were also stained with PAX8. The intensity of staining in the latest varied from 1+ up to 3+. They proposed that the staining of ES/PNET with PAX8 is not dependent on the site of origin [31]. Paul Weisman et al. had recently reported a case of intra-abdominal PAX8 positive ES. The diagnosis was confirmed by the presence of the EWSR1-ERG fusion transcript on RT-PCR testing [32]. In our review, we have found a mild to moderate nuclear positive expression of PAX8 in 12 cases (80%).

## 5. Conclusions

ES is infrequent in general population. The correct diagnosis needs a multidisciplinary approach. The radiological examination plays a fundamental role due to the information provided by CT or MRI as the size, the exact situation, and some aggressiveness radiological criteria as tumoral necrosis or osteolytic damage. In our study, all the cases originated on long bones showed osteolytic behaviour.

From the pathologist's point of view, the diagnosis of ES supposed a challenge because this requires the integration of the morphologic and microscopic criteria with the clinical and radiologic data. Immunohistochemistry supposed as a valuable tool that can help for diagnostic approaching and the differential diagnosis of ES.

In the immunohistochemical analysis, we found that 100% and 80% of the cases showed intense CD99 and mild to moderate PAX8 immunoreactivity, respectively. This could be considered as a high sensitivity even when our sample was not so extended. Nevertheless, CD99 and PAX8 specificity is limited in ES because its immunoreactivity is commonly observed in many other tumours. A complete differential diagnosis is required to rule out other round cell tumours of mesenchymal, lymphoid, or epithelial origin that can also be immunoreactive for these markers. Immunohistochemical and molecular panels are necessary in almost all cases of ES to confirm the diagnosis.

The ES diagnosis must be confirmed by molecular tests. FISH and RT-PCR as well as DNA sequencing by NGS should be performed if possible, in all cases.

This way requires a deeper molecular investigation with a large prospective series to shed light about the implications of PAX8 in the oncogenesis of ES and its potential consequences on therapeutic response and prognosis.

## Figures and Tables

**Table 1:** Clinical and radiological data.

Patient	Gender/age	Primary and metastatic sites	Radiological characteristics
1	M/14	Perone and soft tissue	10 × 6 × 6 cm, lytic
2	M/14	Femur and soft tissue	5 × 4 × 3 cm, lytic
3	F/14	Humerus	18 cm in larger diameter, lytic
4	M/15	Femur and soft tissue	8 × 6 × 5 cm, lytic
5	M/17	Pulmonary metastasis	Metastatic nodes
6	M/19	Tibia	4 × 3 × 3 cm, Lytic
7	F/20	Perineal region	12 × 8,7 × 6,7 cm, Infiltrative
8	F/24	Pulmonary metastasis	Metastatic nodes
9	M/29	Femur and soft tissue	12 cm in larger diameter, lytic
10	F/35	Thigh (soft tissue)	11,5 × 11 × 7,5 cm, infiltrative
11	M/39	Retroperitoneum	12 × 11 × 9,7 cm, infiltrative
12	F/39	Femur and soft tissue	15 cm in larger diameter, lytic
13	M/54	Chest wall	13 × 12 × 3 cm, infiltrative
14	M/60	Gluteus region	5 × 3,9 × 3,4 cm, infiltrative
15	M/73	Lumbosacral spine	5 × 4 × 3 cm, lytic

**Table 2:** Immunohistochemistry methods.

Immunohistochemical markers	Source	Clone	Dilution	Bond^™^ automated system
CD99	Leica	PCB1	1 : 50	Leica Biosystems
PAX8	Master Diagnostics	MRQ-SO	Prediluted	Bond™ Polymer Refine Detection

**Table 3:** The immunoreactive score (IRS).

A (percentage of positive cells)	B (intensity of staining)	IRS score (multiplication of A and B)
0 = no positive cells	0 = no color reaction	0-1 = negative
1 ≤ 10% of positive cells	1 = mild reaction	2-3 = mild
2 = 10–50% positive cells	2 = moderate reaction	4–8 = moderate
3 = 51–80% positive cells	3 = intense reaction	9–12 = strongly positive
4 ≥ 80% of positive cells		

**Table 4:** Immunohistochemistry and FISH results.

Cases	Immunohistochemistry		FISH. *EWSR1* ((%) of cells with rearrangement)
	CD99	PAX8	
1	Strongly positive	Mild	92
2	Strongly positive	Negative	85
3	Strongly positive	Moderate	60
4	Strongly positive	Mild	94
5	Strongly positive	Negative	87
6	Strongly positive	Moderate	75
7	Strongly positive	Moderate	92
8	Strongly positive	Moderate	80
9	Strongly positive	Mild	95
10	Strongly positive	Mild	65
11	Strongly positive	Mild	41
12	Strongly positive	Mild	23
13	Strongly positive	Mild	80
14	Strongly positive	Mild	62
15	Strongly positive	Negative	26

## Data Availability

Data files are publicly available in Harvard Dataverse: https://doi.org/10.7910/DVN/CDIMTD.
